# The Overall Environmental Load and Resistance Risk Caused by Long-Term Fungicide Use to Control *Venturia inaequalis* in Apple Orchards in Latvia

**DOI:** 10.3390/plants12030450

**Published:** 2023-01-18

**Authors:** Regīna Rancāne, Alma Valiuškaitė, Viktorija Zagorska, Vitālijs Komašilovs, Neringa Rasiukevičiūtė

**Affiliations:** 1 Lithuanian Research Centre for Agriculture and Forestry, Institute of Horticulture, Kaunas District, LT-54333 Babtai, Lithuania; 2 Institute for Plant Protection Research “Agrihorts”, Latvia University of Life Sciences and Technologies, LV-3004 Jelgava, Latvia; 3 Faculty of Information Technologies, Latvia University of Life Sciences and Technologies, LV-3001 Jelgava, Latvia

**Keywords:** apple scab, Pesticide Load Indicator, decision support system RIMpro

## Abstract

Apple orchards are perennially planted where pesticides are applied to control numerous pests and diseases. The extensive long-term use of fungicides can lead to overall environmental load and resistance risk. This study aims to assess which fungicide-active substances have been used more intensively in the last decade in Latvia, evaluating the overall environmental load using the Pesticide Load Indicator (PLI). It was essential to see whether the amount of active substance usage rises, how it correlates with the total changes of the PLI and which substances are with the highest scores. The other issue was to test the sensitivity of *Venturia inaequalis* populations to systemic fungicides. Six full-bearing apple orchards that reflected local plant protection practices were selected from the different growing regions of Latvia to analyze fungicide use from 2012 to 2021 and test *V. inaequalis* populations’ sensitivity to systemic substances difenoconazole and cyprodinil. The PLI demonstrated that the protective fungicides were the most crucial group overall, with the highest potential impact on the environment and human health. Systemic fungicides had a relatively lower environmental impact, but after long-term use, the pathogen population’s sensitivity to difenoconazole and cyprodinil was reduced. Introducing new fungicide classes and biological control agents could help growers improve plant protection strategies against *V. inaequalis*, reducing the risk of resistance and environmental load.

## 1. Introduction

Using chemical pesticides in agriculture contributes to soil, water and air pollution and biodiversity loss and can harm non-target plants, insects, birds, mammals and amphibians [1]. Recently, European policies targeted decreasing pesticide usage in all agricultural systems to reduce environmental hazards and health risks. The challenge is exceptionally high in apple orchards, where pesticides are recurrently applied to control numerous pests and diseases [2]. The highest consumption of pesticides is required to control apple scabs, caused by *Venturia inaequalis* (Cookel) G. Winter, the essential apple disease, causing economic losses in many apple production areas, including the Baltic region [3,4]. In the Baltic region, 6–12 fungicide treatments were done per season [4,5], while in Central and Southern Europe, up to 20 treatments may be applied against the *Venturia inaequalis* population in each growing season [6,7]. In 2009, the European Union, by Directive 128/2009EC, established a framework for EU actions to promote the sustainable use of pesticides and determine one of the integrated pest management (IPM) principles to monitor harmful organisms [8]. Therefore, collecting meteorological data and forecasting equipment in orchards are essential in detecting *V. inaequalis* infection risk periods [6]. There are currently several simulation models available for assessing *V. inaequalis* primary infection risks [9,10], for instance, APPLESCAB [11], Ag-Radar [12], WELTE [13], A-scab [10] NEWA [14] and SkyBit [15] whose provide information for infection development based on climatic conditions. In Lithuania, the internet-based system iMETOS^®^sm has been used in practice since 2007 [4]. The decision support system (DSS) RIMpro [16] is the most commonly used in Europe at present [6]. RIMpro has been tested in Latvia to detect infection risks since 2003 [5] and was introduced in practice in 2007 on the most prominent apple farms. Based on weather data, RIMpro helps growers by showing the development of the infection in real-time, estimating the coverage remaining from previous fungicide applications, and the curative period of some fungicides [17].

Fungicides from around 13 chemical or biological groups are registered for use on apples for *V. inaequalis* control in the EU [18], from 10 groups in Lithuania [19] and only eight groups are available in Latvia [20]. Up to 85% of applications are done by protective fungicides, mainly using products containing active substances (a.s.), dithianon (quinones), captan (phthalimides), mancozeb (dithiocarbamates), and copper (II) hydroxide (inorganic). Considering that apple orchards are perennial plantings that grow for at least two decades in one place, the extensive (multiple applications and high doses) use of protective fungicides in orchards can lead to environmental fate. Over the past decade, public concern about the possible side effects of pesticides on human health and the environment has been increasing, and there is a need to assess the risks of using different substances. The disease infection risk indicators are easy-to-use tools that can aid in minimizing the off-site impacts of pesticides and assist in decision-making and policy formulations [21,22]. Researchers used an early treatment frequency index (TFI) to assess the intensity of pesticide use in orchards [2]. TFI expresses the frequency of pesticide treatments. However, they are not considered active ingredient compound toxicity; therefore, they cannot be considered a risk indicator. Danish authorities developed a pesticide risk indicator that could monitor pesticide load and set quantitative targets in response to adverse pesticide impact by EU Directive 128/2009/EC implementation. Hence, a new indicator is named the Pesticide Load Indicator (PLI) [23]. PLI is usually used to identify, in general, which group of pesticides has the highest impact on the environment and human health and to find out the active substance with the highest total effect per country [24]. 

One way to reduce the number of protective treatments would be to use systemic fungicides when there is an increased infection risk. However, plant protection based on system preparations is not recommended due to the resistance developed by the pathogen to individual single-site fungicide groups [25]. According to the FRAC Code List, systemic active substances from the group’s aniline-pyrimidines (AP) and demethylation inhibitors (DMI) have a medium resistance risk [26]. Since *V. inaequalis* has a high risk of developing resistance to fungicides [27], there might be a risk that after long-term use of the same active substance fungicides, the sensitivity of the pathogen to individual active substances may have been decreased. Therefore, in the case of fungicide resistance, ineffective use of fungicides only could lead to additional environmental fate.

This study aims to assess which substances have been used more intensively in the last decade to control apple scab pathogen *V. inaequalis*; determine overall environmental load by using the Pesticide Load Indicator; and test the sensitivity of *V. inaequalis* populations to systemic substances difenoconazole and cyprodinil, taking into account the last 10 year of spraying data.

## 2. Materials and Methods

### 2.1. Orchards Description and Apple Scab Management 

Six full-bearing apple orchards of similar age, typically to reflect local plant protection practices, were selected from the different growing regions of Latvia to analyze fungicide use from 2012 to 2021 (Table 1) and collect *V. inaequalis* populations. As reported in Table 1, many different cultivars have been selected: Auksis (all farms), Alva (Farm 1), Ligol (Farm 1 and 6), Sinap Orlovskij (Farm 1, 2, 3, 4 and 5), Belorusskoje Malinovoje (Farms 2, 3, 4 and 6) and Lobo (Farms 2, 3 and 6). Since 2007, the commercial platform DSS RIMpro [5,16,17,28] was employed in all six farms to predict apple scab primary infection risks and set up fungicide applications. Meteorological stations Lufft (G. Lufft Mess and Regeltechnik GmbH, Fellbach, Germany) equipped with air and soil temperature, precipitation, leaf wetness, relative humidity, and solar radiation sensors were placed in each orchard. Decision support system (DSS) RIMpro predicts apple scab infection risks based on real-time and weather forecast data. As RIMpro is a commercial platform, detailed model algorithms are not publicly available. RIMpro provides forecasting to control apple scabs. The DSS by RIMpro could be (1) preventive treatments before the rain, during the germination window, or (2) a curative treatment after the infection risk. The application type depends on infection risk and the used fungicide. Two treatments are recommended during extremely high infection events: a preventive treatment shortly before the rain and a curative treatment during the germination window [17]. However, it should be noted that the spraying intensity differed among orchards (Table 1) and did not always correspond to the number of infection risks. Trees in orchards were on B 118 or M 26, MM 106 rootstocks and pruned to a pyramidal shape. Tree density ranged from 660 to 1200 plants per hectare. 

### 2.2. Pesticide Risk Assessment

To calculate the quantity of active substances used to control scabs and to estimate the Pesticide Load Indicator (PLI), data on fungicide consumption reported by six apple growers from the 10 growing seasons (2012–2021) were used (Table 2). 

Each year, the number of application times and amount of use in kilograms on each farm were counted for each active substance. In addition, considering that growers also use fungicide mixtures by spraying two substances simultaneously, the frequency of active substances used was also counted. 

PLI [23] consists of three sub-indicators: human health (PL_HH_), ecotoxicology (PL_ECO_) and environmental fate (PL_FATE_). PL_ECO_ and PL_FATE_ were calculated and expressed as the PLI per unit (kg or L) of the active substance, and PL_HH_ were based on the risk phrases of active substances, not the product, as it was according to Danish methodology [23]. The decision was made due to the evaluation of active substance toxicity, not the active component toxicity, avoiding the case when some co-formulants can be determining factors [29]. 

PL_ECO_ is determined using lethal concentration (LC50), lethal dose (LD50), half maximal effective concentration (EC50) and no observable effect level (NOEC) values from the Pesticide Property Database (PPDB) [30]. PL_FATE_ is made up of three input parameters, the half-life in soil (DT50) in laboratory conditions, the bioaccumulation factor (BCF) (or the log Pow value if no BCF value is reported) and the screening concentration in groundwater (SCI-GROW index) [31] that reflects the mobility and risk of leaching to the groundwater of the active substance and the significant metabolites listed in the PPDB database [30].

According to Danish methodology [23], the year 2011 was taken as the starting point for the reference values in PL calculation. Therefore, for both PL_ECO_ and PL_FATE,_ the most harmful pesticide active substances registered in Latvia from the year 2011 within each input parameter (lowest LC50, LD50, EC50 and NOEC values, longest DT50 in lab, highest BCF and SCI GROW index) have been defined as the reference active substances and is allocated the maximum number of PL points per kg active substance (Table 3).

For PL_HH_, every risk phrase for an active substance scored between 10 and 100. The highest score (100 points) is provided to highly toxic products that can cause irreversible damage (e.g., heritable genetic risks or cancer), while skin irritation or respiratory irritation only generates 10 points [23].

### 2.3. Fungicide Resistance Assays

The apple leaves samples with scab lesions were collected in 2020 and 2021 (Table 4) to verify resistance development to difenoconazole and cyprodinil active ingredients. One sample (100 leaves) with actively sporulating scab lesions was collected from each orchard. 

The leaves were dried in an open container for one week. The dried samples were provided to Bio-Protect GmbH (Konstanz, Germany) to evaluate the sensitivity of the fungus by in vivo method on potted apple trees. Samples were frozen and stored at −20 °C until analysis. Before preparing a conidial suspension, 8 g of the sample was thawed and washed in 80 mL of tap water. After counting conidia with a hematocytometer, the suspension was filtered through mull and adjusted to 10^5^ mL^−1^. The suspension was sprayed on the four youngest unfolded leaves of growing shoots of potted apple plants of the cultivar Jonagold grafted on M9 rootstocks grown in a greenhouse. Cultivar Jonagold was selected because it is highly susceptible to *V. inaequalis.*

Five shoots were used per treatment, including untreated control, which was not sprayed with conidia suspension. After plant inoculation, they incubated in a dew chamber at 15–25 °C for 20 h to allow germination of the conidia and infection of the leaves. The curative application was made 24 h after inoculation by spraying the fungicide suspensions until runoff. The fungicide assays were performed at two concentrations: the recommended rate for apple scab treatment and a reduced dose. Difenoconazole was tested at 37.5 and 3.75 mg L^−1^ using Score^®^ 250 EC (Syngenta Agro GmbH, Frankfurt am Main, Germany) containing 250 g/L (23.6% *w/w*) of the active substance. Cyprodinil (Chorus^®^ 50 WG Syngenta Agro GmbH, 500 g kg^−1^) was tested at 150 and 50 mg L^−1^. After the application, plants were incubated in the greenhouse (minimum 12 °C and 12 h light) until symptoms developed in untreated plants (14–28 days). The severity of apple scab symptoms per leaf was rated as the area covered with sporulating lesions. The efficiency of the tested compound was calculated according to Abbott [32]. Tests were done twice for each sample on five shoots per test, and the efficacies from up to 10 shoots (replicates) were averaged and plotted in a dose–response relationship compared to the baseline sensitivity. Baseline sensitivities to difenoconazole [33] and cyprodinil [34] were established before the introduction of the two active substances in Germany in the 1990ies by testing apple scab populations without fungicide history. An apple scab population from an untreated orchard (cultivar Jonagold) from Konstanz, Germany, was multiplied several times in greenhouse experiments without selection pressure since 2005 and used as a sensitive standard. The resistant standards are derived from German orchards with fungicide history [33,34]. 

### 2.4. Statistical Analysis

The data were processed, and the standard deviation was calculated using Microsoft Excel 2016 (Microsoft Corporation by Impressa Systems, Santa Rosa, CA, USA). Box plots were created using Matplotlib 3.6.2 (Matplotlib Development Team) documentation. 

## 3. Results

### 3.1. Long-Term Fungicide Use in Apple Orchards

**Figure 1:**
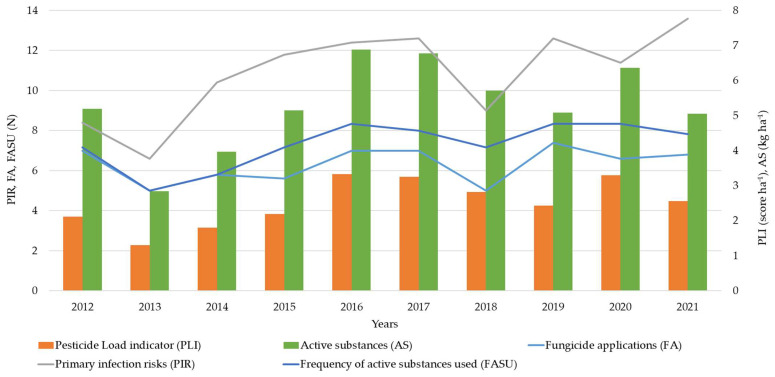
Average number (N) of primary infection risks (PIR), fungicides applications (FA), frequency of active substances used (FASU), Pesticide Load Indicator (PLI) score and quantity of active substances per hectare referred to the six farms considered during 2012–2021 apple-growing seasons.

Fungicides were used during the primary infection period of *V. inaequalis*, characterized by the number of primary infection risks (Figure 1). Our data (Figure 1) indicate a visible interaction between the number of fungicide applications and primary infection risks. Since 2014, there has been an increasing importance of tank mixing, resulting in the frequency of active substances used being more significant than the number of sprays (Figure 1). The annual average number of fungicide applications and frequency of active substances used varied among the years and orchards but generally tended to increase (Table 1, Figure 1). 

**Figure 2:**
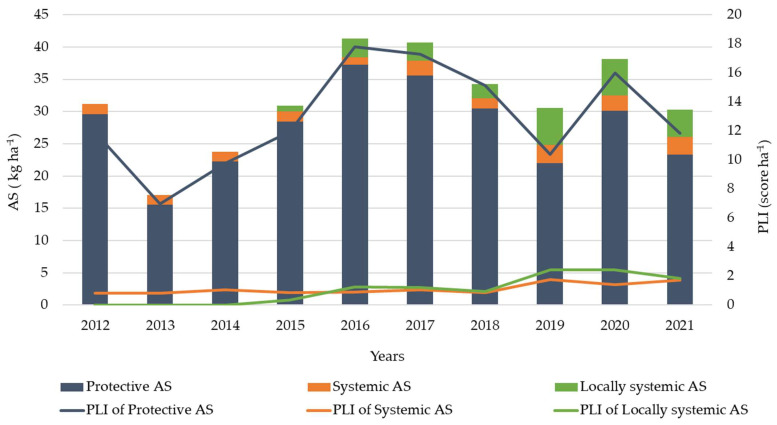
The total consumption of protective, locally systemic and systemic active substances (AS) and Pesticide Load Indicator (PLI) of protective, locally systemic and systemic active substances (AS) per hectare referred to the six farms considered during the 2012–2021 apple-growing seasons.

Our data analysis shows that protective active substances were the essential group (comprising 86% of the overall consumption of active substances), both in kilograms and in PLI scores, followed by locally systemic and systemic active substances (Figure 2). Since 2015, growers have begun using locally systemic fungicides with active substances dodine and kresoxim-methyl, which had not been used before (Table 2). Furthermore, in the last three years, the use of systemic active substances increased because fungicides containing two active substances—dithianon and potassium phosphonate replaced a product that contained only dithianon (Table 2, Figure 2).

### 3.2. Fungicide Risks on Human Health, Ecotoxicology and Environmental Fate

The evaluation of PLI per kg showed that the highest points got active substances difenoconazole (1.26), potassium phosphonate (1.05) and captan (0.84) (Table 5). The other fact worthy of attention is that active substances of the protective fungicides gave more points due to their high dose compared to systemic active substances. For example, applying captan at a maximum dose of 2.25 kg per ha—PLI was 1.51, while difenoconazole with a higher PLI score per kg—1.26, at a maximums dose, gave only 0.06 points (Table 5). An exception to systemic preparations was potassium phosphonate, which had both high PLI per kg and high doses per hectare.

**Figure 3:**
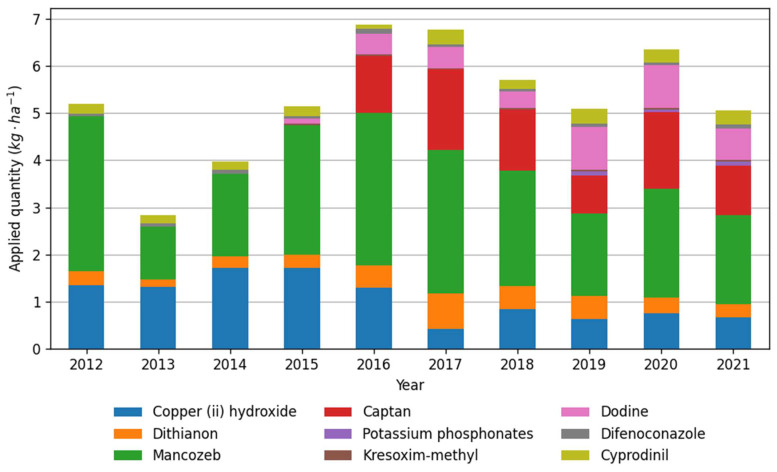
Active substances applied in the selected six farms during the 2012–2021 apple-growing seasons. Data are the means of the consumption of active substances in six farms.

**Figure 4:**
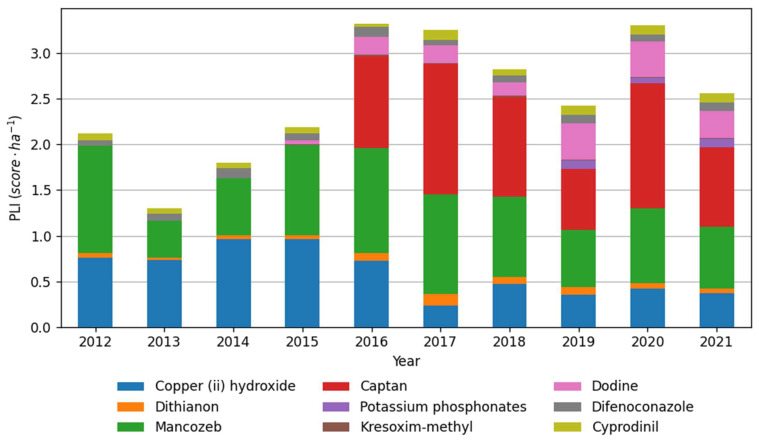
The average Pesticide Load Indicator (PLI) score of individual active substances per hectare per six farms during the 2012–2021 apple-growing seasons.

**Figure 5:**
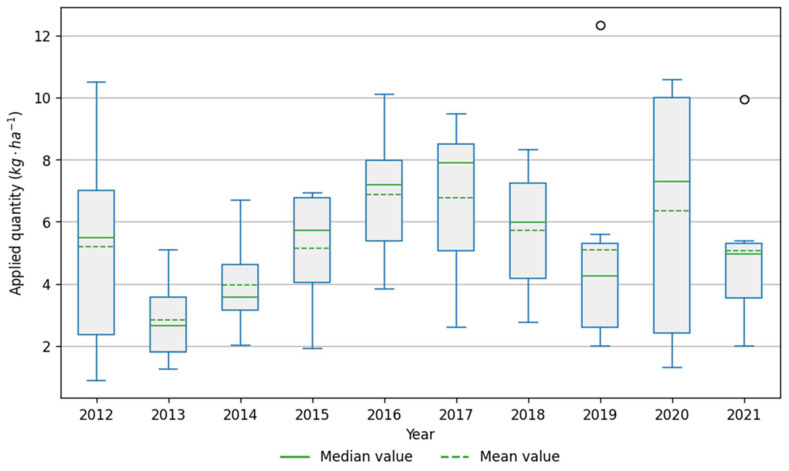
Distribution of data on the use of active substances in the selected six farms during the 2012–2021 apple-growing seasons.

The analyzed data on long-term fungicide use by farms showed that the most intensively used protective substances in the analyzed period were mancozeb, copper (II) hydroxide and captan (Figure 3). These three active substances also formed the highest PLI per hectare, respectively 0.37, 0.68 and 0.87 points on average (Figure 3 and Figure 4) in the year 2021. The PLI increased in 2016 when captan was registered, and growers began to use it instead of copper (II) hydroxide. As a result, the captan has the highest PLI per kg (0.84) among the protective fungicides registered in apple orchards (Table 5). Combined with relatively large doses of preparations, 1.8–2.25 kg per hectare, it increased the total PLI score (Figure 2). Since 2016, dodine has been broadly used, accounting for about 10% of the average PLI per hectare. Whereas considering that the registered dose of copper fungicide was reduced in 2016 from 3 to 1 kg per hectare, the copper (II) hydroxide consumption decreased in recent five years, and the PLI score correspondingly. The other active substances formed a relatively lower average kilogram and PLI per hectare (Figure 3 and Figure 4). The distribution of data within selected six farms during the 2012–2021 apple-growing seasons is shown in Figure 5. The applied quantity of active substances varied across selected Farms, and outlier points were formed by Farm 1 (Figure 5), which generally stood out with higher consumption of fungicides (Table 1, Figure 5). 

**Figure 6:**
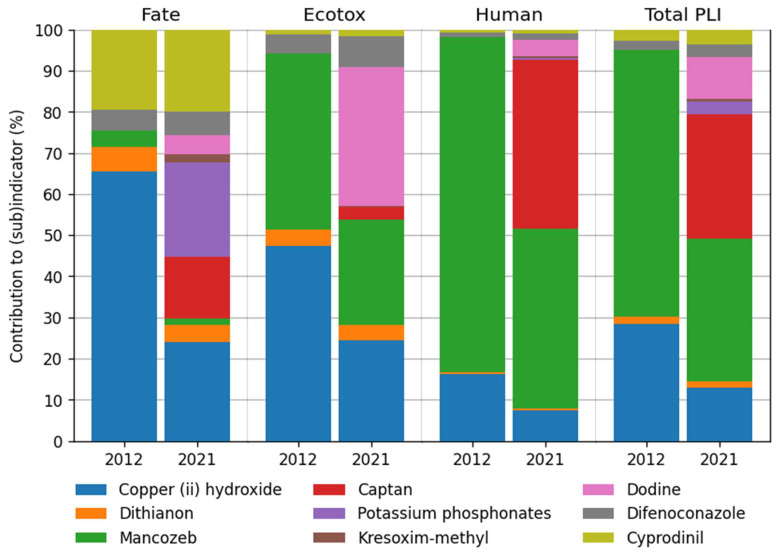
Contribution of active substances to sub-indicators: human health (PL_HH_), ecotoxicology (PL_ECO_), environmental fate (PL_FATE_) and the total Pesticide Load Indicator (PLI) in six farms.

In 2021, the total PLI was formed by nine substances (in 2012—five substances). In 2012 the worst impact on the environment (PL_FATE_) was calculated for the copper (II) hydroxide, but the reduction in the applied maximal dose in 2016 and the introduction of new substances gave the reduction in the copper (II) hydroxide in the year 2021 (Figure 6). In both years, cyprodinil had the same impact of about 20% on PL_FATE_. Potassium phosphonate, which had the highest sub-indicator PL_FATE_ 0.96 per kg, was the most affected environmental fate in 2021 (Table 5, Figure 6)_._ The highest ecotoxicology indicator risk was found for copper (II) hydroxide and mancozeb in 2012 and 2021. In 2021 risk on PL_ECO_ was also made by the active ingredient dodine. The highest risk for human health in 2012 was compiled by mancozeb, but in 2021, the main risk was divided between mancozeb and captan. As the new substance was registered and applied, the usage of mancozeb decreased. Both substances have the highest sub-indicator PL_HH_, respectively, 0.43 and 0.77 points per kg (Table 5, Figure 6). It should be noted that since 2022 mancozeb has been banned in the EU [35], there is a concern that captan use will increase instead, unfortunately leaving a relatively high risk to human health. 

### 3.3. Sensitivity of Venturia Inaequalis Populations to Systemic Fungicides 

Systemic fungicides had a relatively lower environmental impact, but after long-term use, there were concerns about the formation of the resistance. In the orchards included in the study, difenoconazole fungicide applications were carried out on average 1.2–2.5 times (Table 4), at a maximum of four times per season. In Farms 1 and 3, difenoconazole fungicides have been used for all 10 years. The average number of cyprodinil fungicide applications was carried out 1.3–2.0 times (Table 4), with a maximum of three times per season. In Farm 6, cyprodinil fungicides have been used for all 10 years.

Performing resistance testing in sensitive samples, the application of 37.5 mg L^−1^ difenoconazole or 150 mg L^−1^ cyprodinil resulted in >95% control and 3.75 mg L^−1^ difenoconazole or 50 mg L^−1^ cyprodinil resulted in >90% control. The difenoconazole and cyprodinil efficacy against the sensitive reference was comparable to the baseline sensitivity in this study (Table 6). Sensitivities of the samples from Farms 1, 4 and 6 to difenoconazole at the highest dose were reduced (Table 6) compared to the baseline sensitivities. The highest efficacy (97%) was reached against the sample from Farm 3 using 37.5 mg L^−1^ difenoconazole fungicide. Sensitivities of the sample from Farms 1, 2 and 6 to cyprodinil at the highest dose were significantly reduced (Table 6) compared to the baseline sensitivities. The highest efficacy (98%) was reached against the sample from Farm 3 using 150 mg L^−1^ cyprodinil fungicide. 

## 4. Discussion

Apple orchards are perennial plantings that grow for at least two decades in one place. The extensive (multiple applications and high doses) use of fungicides in orchards can lead to environmental fate. An inventory of fungicide use shows intensity but does not reflect the environmental impact. Our case study showed that the Pesticide Load Indicator (PLI) is a helpful tool to demonstrate which group of active substances has the highest impact on the environment and human health. Active substances registered for apple scab control in Latvia copper (II) hydroxide, mancozeb, dithianon, cyprodinil and difenoconazole have been used for all 10 years, while a.s. captan, dodine, kresoxim-methyl and potassium phosphonate were registered and started to be used later. The analyzed data showed that since 2015, farmers started to use fungicides more intensively as orchards became “older” and the inoculum pressure increased.

Protective fungicides were the essential group overall, forming the highest PLI. The highest risk for human health was expected by captan, even higher than for mancozeb, which has just been fixed for the non-renewal of the authorization as an endocrine disruptor [35]. This means that captan will be used more intensively to replace mancozeb in the future, contributing to PLI growth. The dithianon with low PLI has shown promising results in preventing apple scabs, but in the last years on the market, there was only a fungicide containing two substances, dithianon and potassium phosphonate led to the PLI rise. In general, potassium phosphonate is controversial, and some studies refer to it as an effective fungicide against various pathogens, which in combined treatments, reduces the need for traditional fungicides [36,37]. However, others point out that increasing potassium phosphonate persistence in soil may impart selective pressure on fungal resistance mechanisms, negatively influencing symbiotic relationships between plants and mycorrhizal fungi [37]. In Denmark and Norway, pesticides are placed in tax classes differentiated according to health and environmental factors [38]. According to the tax system in Norway, fungicides are classified into six tax classes, which means that the higher the number (0–6), the higher the tax, and fungicides containing potassium phosphonate are in tax class 5 [39]. Dodine, whose mode of action is mentioned as locally systemic, and protective, is registered to use only two times per season and has a 60-day pre-harvest interval. Therefore, it also cannot wholly replace protective fungicides. Kresoxim-methyl, the active substance of a locally systemic fungicide, is also recommended more as a protective fungicide according to RIMpro forecasts but is rarely used by growers due to the high risk of resistance [26]. Unfortunately, there are no other equivalent alternatives for protective applications. It should be added that copper (II) hydroxide is no longer officially registered for apple scab control as of 2016, but growers still use it as the first protective fungicide in spring for the first application.

Regarding copper, raised the discussion on how to evaluate the PLI of copper (II) hydroxide because, according to Danish methodology, parameter soil degradation DT50 in the lab is needed to calculate sub-indicators for environmental fate (PL_FATE_). This value is not available in the PPDB database. Danish methodology suggests giving 0 points for this parameter as copper is an inorganic substance of natural origin. However, this contradicts other studies that state copper cannot be degraded, and its removal from the soil is negligible through leaching, runoff or plant uptake. Therefore, this heavy metal can potentially contaminate the environment for long periods and cause bioaccumulation and toxicity [40]. The long decomposition time of copper is also confirmed by the PPDB DataBase, where it is stated that soil degradation DT50 (field) is 2600 days, which is a very long period. For instance, potassium phosphonate has higher PL_FATE_, although DT50 (field) is 142 days. Since the accumulation of copper is hazardous to micro and macroorganisms, and microorganisms are generally more sensitive to copper than other organisms in soil biocoenosis [40], copper fungicides are banned in Scandinavian countries and the Netherlands. In Latvia, the consumption of copper preparations has been relatively low in orchards in the last decade, especially after the Bordeaux mixture was removed from the register and copper (II) hydroxide dose was reduced to 1 kg per hectare per one application since 2016. The above indicates that the methodology of PLI calculations for both copper should be revised further. 

Systemic fungicides containing difenoconazole and cyprodinil are available for growers. Although the PLI of these active substances is relatively low due to small doses, intensive use of these preparations is not recommended due to the risk of resistance. Summarizing the results of the fungicide sensitivity tests, we found a strong correlation between the effectiveness provided by cyprodinil and the number of fungicide treatments by year—as the number of treatments increased, the effectiveness decreased. A decrease in pathogen sensitivity to difenoconazole was found in several orchards. It can be concluded that there is a limited choice of active substances in Latvia. Therefore, applying a strategy that would be environmentally friendly, effective and without the risk of resistance is complex. The introduction of new fungicide classes, e.g., succinate dehydrogenase inhibitors (SDHIs, group 7) and control agents (BCAs), could help growers to improve plant protection strategies against the apple scabs, both reducing the risk of resistance and improving efficacy [41]. Biological fungicides would help reduce the impact on the environment, but unfortunately, biological control agents reduce *V. inaequalis* population rather than control them and may not be effective enough for use during the growing season [42]. Alternative products such as biological control agents, biostimulants and plant extracts for scab management are often tested in the laboratory. However, biological products are still not widely used in the industry because the disease control they offer is frequently unpredictable and they typically require more applications than conventional chemicals do. The best solution so far would be to incorporate synthetic and soft fungicides in an alternating spray schedule [42]. In this case, using DSS is significant for determining the precise timing of spraying each active substance.

## 5. Conclusions

Analyzing our long-term fungicide usage data, it can be seen that protective fungicides were the most crucial essential group overall, both in kilograms and in PLI s, causing the potential impact on the environment. There was a considerable difference in kg applied for protective fungicides from 29.61 to 37.25 kg per ha, and for systemic from 1.15 to 2.80 kg per ha applied, there is a difference of more than 20 times in applied volumes and PLI correspondingly. Systemic fungicides had a relatively lower PLI per kg applied and PLI per dosage applied, but the long-term use of a few systemic fungicides reduced the pathogen population’s sensitivity to difenoconazole and cyprodinil was detected. The main factors leading to the sensitivity reduction were the same active substance usage per season more than two times during more than five years. Following the green deal, many active substances will be banned, thus increasing the risks of using the remaining substances. It is expected that if no new substances are registered, the resistance will grow; if no new substances are registered, the resistance will grow, and there will be a need for a new solution search for apple orchard protection. It should be stated that an average Latvian apple orchard spraying schedule is considered to be environmentally friendly. Growers are taking into account the decision support system recommendations but the number of fungicide treatments is lower than the system recommends, resulting in lower fruit quality than required. Taking into account the need to reduce pesticide use by 50% by the year 2030, it can be stated that this will lead to even lower quality and higher prices of Latvian apples, as the only available alternative will be sanitation measures to reduce the incidence level of the pathogen population. The other way could be the introduction of scab-resistant varieties, but it is a long and gradual process.

## Figures and Tables

**Table 1:** Location and characteristics of apple orchards used in this study.

District	Region	Farms	Planting Year	The Main Cultivars	Annual Applications *
Bauska, GPS 56.371554, 24.279826	South	Farm 1	2000	Auksis, Alva, Ligol, Sinap Orlovskij	8.7 ± 1.83
Jēkabpils, GPS 56.274396, 25.396625	South	Farm 2	1999	Auksis, Belorusskoje Malinovoje, Lobo, Sinap Orlovskij	5.2 ± 1.23
Talsi, GPS 57.396865, 22.941191	North	Farm 3	2000	Auksis, Belorusskoje Malinovoje, Lobo, Sinap Orlovskij	6.7 ± 1.77
Balvi, GPS 57.226005, 27.678585	North	Farm 4	2000	Auksis, Belorusskoje Malinovoje, Sinap Orlovskij	4.6 ± 1.17
Sigulda, GPS 57.132812, 24.854656	Central	Farm 5	2000	Auksis, Kovalenkovskoje Saltanat, Sinap Orlovskij	4.6 ± 0.84
Valmiera, GPS 57.582202, 25.107896	Central	Farm 6	2002	Auksis, Belorusskoje Malinovoje, Ligol, Lobo	8.0 ± 1.89

* Data are shown as a mean (*n* = 10) ± standard deviation.

**Table 2:** Fungicides used against *Venturia inaequalis* during the 2012–2021 growing seasons.

Active Substances	Group Name *	Activity **	Mode of Action *	Years of Use
copper (II) hydroxide	Inorganic	Preventive	multi-site contact activity	2012–2021
dithianon	Quinones		multi-site contact activity	2012–2021
captan	Phthalimides		multi-site contact activity	2016–2021
mancozeb	Dithiocarbamates		multi-site contact activity	2012–2021
kresoxim-methyl	QoI-fungicides	Locally systemic	respiration	2015–2021
dodine	Guanidines		unknown mode of action	2015–2021
difenoconazole	DMI-fungicides	Systemic	sterol biosynthesis in membranes	2012–2021
cyprodinil	AP-fungicides		amino acids and protein synthesis	2012–2021
potassium phosphonate	Phosphonates		host plant defence induction	2019–2021

* Group names and mode of action are shown accordingly to FRAC Code List ©*2022 [26]. ** Activity is shown to List of plant protection products registered in Latvia [20].

**Table 3:** Parameters included in the calculation of the Pesticide Load Indicator.

Input Parameters	Reference Active Substance	Unit	Maximum Value	PL Points
Ecotoxicology				
Birds—acute LD50	Methiocarb	mg kg^−1^ body weight	5	1
Mammals—acute oral LD50	Aluminium phosphide	mg kg^−1^ body weight	8.7	1
Fish—acute 96 h LC50	Gamma-cyhalothrin	mg L^−1^ water	0.000035	30
Daphnia—acute 48 h EC50	Gamma-cyhalothrin	mg L^−1^ water	0.000045	30
Algae—acute 72 h EC50	Bifenox	mg L^−1^ water	0.00018	3
Aquatic Plants—7 day EC50	Triasulfuron	mg L^−1^ water	0.000068	3
Earthworms—14 day LC50	Beta-cyfluthrin	mg kg^−1^ soil	0.565	2
Honeybees—acute 48 h LD50	Deltamethrin	mg bee^−1^	0.0015	100
Fish—chronic 21 day NOEC	Alpha-cypermethrin	mg L^−1^ water	0.00003	3
Daphnia—chronic 21 day NOEC	Gamma-cyhalothrin	mg L^−1^ water	0.0000022	3
Earthworms—chronic 14 day NOEC	Dimoxystrobin	mg kg^−1^ soil	0.089	2
Environmental fate				
Soil degradation—DT50	Diquat	days	2345	2.5
Bioaccumulation	Metaflumizone	bio-concentration factor	7800	2.5
Mobility—SCI-GROW index	Flutriafol	SCI-GROW index	7.09	20
Water DT50	Epoxiconazole	days	1000	-

**Table 4:** The origin and fungicide history of the *Venturia inaequalis* populations used for the resistance testing.

Farms	Cultivar	Sampling Date	Difenoconazole		Cyprodinil	
			Annual Applications *	Years	Annual Applications *	Years
Farm 1	Alva	07.2020	2.5 ± 0.85	10	2.0 ± 0.53	8
Farm 2	Lobo	07.2020	1.4 ± 0.55	5	2.0 ± 0.82	7
Farm 3	Lobo	09.2020	1.4 ± 0.70	10	1.5 ± 0.55	6
Farm 4	Auksis	07.2021	1.2 ± 0.45	5	1.6 ± 0.52	8
Farm 5	Kovalenkovskoje	07.2021	1.6 ± 0.53	9	1.3 ± 0.50	9
Farm 6	Lobo	07.2020	1.6 ± 0.73	9	1.9 ± 0.74	10

* Data are shown as a mean *n* ± standard deviation.

**Table 5:** Pesticide Load Indicator (PLI) sub-indicators: Environmental fate (PL_FATE_), Ecotoxicology (PL_ECO_), Human health (PL_HH_) and total PLI score calculated per unit of the active substance (AS) and maximum dose of selected fungicides.

PLI Sub-Indicators and PLI Scores	Preventive				Locally Systemic		Systemic		
	Copper (II) Hydroxide	Mancozeb	Captan	Dithi-anon	Dodine	Kresoxim-methyl	Potassium Phosphonate	Cyprodinil	Difenoconazole
PL_FATE_	0.13	0.00	0.05	0.06	0.03	0.07	0.96	0.24	0.29
PL_ECO_	0.21	0.06	0.02	0.08	0.29	0.03	0.01	0.03	0.61
PL_HH_	0.22	0.30	0.77	0.03	0.12	0.23	0.08	0.07	0.37
Total PLI per 1 kg of AS	0.56	0.36	0.84	0.17	0.43	0.34	1.05	0.34	1.26
Maximum registered fungicide dose in 2021 (kg ha^−1^)	1.00	2.00	2.25	0.50	1.25	0.20	2.50	0.45	0.20
AS per maximum dose (g)	500	1500	1800	350	680	100	1402.5	225	50
Total PLI per unit of maximum dose of AS	0.28	0.54	1.51	0.06	0.29	0.03	1.47	0.08	0.06

**Table 6:** Baseline sensitivity and efficacy of the difenoconazole and cyprodinil after curative application against sensitive and resistant *Venturia inaequalis* population and populations collected in six farms.

Active Substance Concentration (mg L^−1^)	Baseline Sensitivity [33,34]	*Venturia inaequalis* Populations							
		Sensitive	Resistant	Farm 1	Farm 2	Farm 3	Farm 4	Farm 5	Farm 6
	Efficacy (%)								
Difenoconazole, 37.5	100	100	75	68	90	97	68	93	88
Difenoconazole, 3.75	95	92	15	46	47	49	67	78	45
Cyprodinil, 150	99	100	54	77	80	98	91	90	58
Cyprodinil, 50	93	98	37	26	63	72	94	88	35

## Data Availability

Not applicable.
